# Polyunsaturated Fatty Acids Stimulate *De novo* Lipogenesis and Improve Glucose Homeostasis during Refeeding with High Fat Diet

**DOI:** 10.3389/fphys.2017.00178

**Published:** 2017-03-23

**Authors:** Raffaella Crescenzo, Arianna Mazzoli, Rosa Cancelliere, Francesca Bianco, Antonia Giacco, Giovanna Liverini, Abdul G. Dulloo, Susanna Iossa

**Affiliations:** ^1^Department of Biology, University of Naples Federico IINaples, Italy; ^2^Division of Physiology, Department of Medicine, University of FribourgFribourg, Switzerland

**Keywords:** caloric restriction, polyunsaturated fatty acids, *de novo* lipogenesis, hepatic inflammation, insulin resistance

## Abstract

**Aims:** The recovery of body weight after a period of caloric restriction is accompanied by an enhanced efficiency of fat deposition and hyperinsulinemia—which are exacerbated by isocaloric refeeding on a high fat diet rich in saturated and monounsaturated fatty acids (SFA-MUFA), and poor in polyunsaturated fatty acids (PUFA), and associated with a blunting of *de novo* lipogenesis in adipose tissue and liver. As high fat diets rich in PUFA have been shown to limit the excess fat deposition and improve glucose homeostasis, we investigated here the extent to which *de novo* lipogenesis in liver and adipose tissues (white and brown), as well as hepatic oxidative stress, are influenced by refeeding on diets rich in PUFA.

**Design:** In rats calorically restricted for 14 days and refed for 14 days on isocaloric amounts of a high fat diet rich in lard (i.e., high SFA-MUFA) or in safflower and linseed oils (rich in PUFA), we investigated energy balance, body composition, glycemic profile, and the regulation of fatty acid synthase (rate-limiting enzyme of *de novo* lipogenesis) in liver, white and brown adipose tissue. We also evaluated oxidative stress in liver and skeletal muscle and markers of hepatic inflammation.

**Results:** Rats refed the PUFA diet gained less lipids and more proteins compared to rats refed SFA-MUFA diet and showed lower amount of visceral and epididymal white adipose tissue, but increased depots of interscapular brown adipose tissue, with higher expression of the uncoupling protein 1. A significant increase in non-protein respiratory quotient and carbohydrate utilization was found in rats refed PUFA diet. Rats refed PUFA diet showed improved glucose homeostasis, as well as lower triglycerides and cholesterol levels. Fatty acid synthase activity was significantly higher in liver, white and brown adipose tissue, while lipid peroxidation and the degree of inflammation in the liver were significantly lower, in rats refed PUFA diet.

**Conclusions:** When considering the composition of high fat diets for nutritional rehabilitation, the inclusion of PUFA could be useful for improving protein deposition and maintaining glucose homeostasis, while limiting lipid storage in adipose tissue and oxidative stress and inflammation in the liver.

## Introduction

The World Health Organization Consultation on Obesity concluded that behavioral and environmental factors have been, during the past two decades, primarily responsible for the dramatic increase in obesity, a condition of energy imbalance and excessive fat deposition (Racette et al., 2003). In Western society, characterized by sedentary lifestyle combined with excess energy intake, the global incidence of obesity continues to increase and, as a consequence, many people often try to lose weight with caloric restriction. However, it is well known that the weight regain is common after caloric restriction, and that it is accompanied by an accelerated fat storage in adipose tissue (Dulloo et al., 2006). The high efficiency of recovery of the energy reserves of body fat probably evolved in ancient times, when the food availability was intermittent and it was necessary to prepare for long periods of famine. Nowadays, it is a key factor causing higher body fat gain relative to lean tissue and this preferential catch-up fat phenomenon has also been linked to the hyperinsulinemic state of catch-up growth and the associated risks for later development of the metabolic syndrome (Crescenzo et al., 2003; Dulloo et al., 2006). Several studies of refeeding after caloric restriction have been conducted on laboratory rats, in which energy intake, diet composition and the level of physical activity can be easily monitored with high accuracy. Furthermore, considering the standard housing conditions, laboratory rats exhibit a sedentary behavior, similarly to what happens in humans (Spangenberg et al., 2005; Buettner et al., 2007; Aydin et al., 2014). In previous studies (Crescenzo et al., 2003; De Andrade et al., 2015), we observed that after a period of caloric restriction, when rats are refed with a low fat diet, they exhibit a reduction in energy expenditure and an increase in metabolic efficiency that give rise to an elevated rate of body fat deposition (even in absence of hyperphagia), together with a reduced insulin sensitivity (Crescenzo et al., 2003; Cettour-Rose et al., 2005). The above metabolic alterations are exacerbated by refeeding on a high fat diet (Dulloo and Girardier, 1992; Crescenzo et al., 2003, 2012; Marcelino et al., 2013). Indeed, it was shown that glucose homeostasis is impaired during refeeding with high fat diets rich in lard, mainly containing saturated fatty acids (SFA) and monounsaturated fatty acids (MUFA), partly because the pathway of *de novo* lipogenesis is markedly inhibited in liver and white adipose tissue (WAT) (Marcelino et al., 2013).

In the light of previous findings showing that the phenomenon of catch-up fat and the degree of insulin resistance during refeeding on a high fat (lard) diet can be ameliorated using high fat diets rich in safflower/linseed oil (polyunsaturated fatty acids (PUFA) of omega-6 and omega-3 series) (Dulloo et al., 1995; Yepuri et al., 2011), we investigated here the extent to which these metabolic improvements could be related to the regulation of *de novo* lipogenesis by these dietary fatty acids. To this end, we investigated here the regulation of the pathway of *de novo* lipogenesis by the different fatty acids in liver, WAT, and brown adipose tissue (BAT) using a high fat diet, rich in safflower/linseed oil (PUFA diet) or a high fat diet rich in lard (SFA-MUFA diet), given to rats during refeeding after a period of caloric restriction. We also evaluated mitochondrial activity in liver and skeletal muscle, since both these tissues are major contributors to daily metabolic rate (Rolfe and Brown, 1977) and it has been proposed that skeletal muscle is involved in the suppression of thermogenesis that underlines the high metabolic efficiency for accelerated body fat recovery after caloric restriction (Dulloo, 2005; Crescenzo et al., 2006).

## Materials and methods

### General study design

Male Sprague-Dawley rats, aged 6 weeks, were adapted to room and cage environments for at least 5 days prior to the start of each experiment; they were caged singly in a temperature-controlled room (22 ± 1°C) with a 12-h light/dark cycle. They were maintained on a commercial pelleted chow diet (Mucedola 4RF21; Settimo Milanese, Milan, Italy) consisting, by energy, of 29.0% protein, 60.4% carbohydrates, and 10.6% fat, and had free access to tap water. The experiments were conducted after this period of adaptation in rats selected on the basis of body weight being within ±5 g of the mean body weight (200 g). This study was carried out in accordance with the recommendations of “Italian Health Ministry.” The protocol was approved by the “Comitato Etico-Scientifico per la Sperimentazione Animale” of the University “Federico II” of Naples.

### Experimental design

Rats were food restricted on a daily basis for 14 days at approximately 50% of the spontaneous chow intake (calculated as mean of the food intake per day in the same rats before semistarvation). It has been previously shown that this degree of caloric restriction induces growth arrest, marked depletion of body fat stores, and leads to diminished energy expenditure (due to suppressed thermogenesis) that underlies a high efficiency of fat deposition during refeeding (Crescenzo et al., 2003). At the end of the semistarvation period, all the rats were separated into 3 groups (*n* = 8): one group was immediately euthanized by decapitation to calculate the body composition of restricted rats, while the other two groups were refed isocaloric amounts of two different high fat diets (58.2% by energy), rich in lard (mainly MUFA and SFA) or safflower/linseed oil (PUFA of omega-6 and omega-3 series). The amount of dietary energy provided to the refed animals corresponds to the metabolisable energy intake of spontaneously growing (non-restricted) weight-matched control animals fed on chow diet, as previously reported (Crescenzo et al., 2003). Furthermore, the level of fat in the high fat diet utilized here (i.e., 58% of energy intake) corresponds to dietary fat levels often utilized in rehabilitation (energy-dense) diets of malnourished infants and children in order to meet their high energy requirements for catch-up growth (Prentice and Paul, 2000). Details of diets composition and energy content are reported in Table 1. This experimental design, consisting of 2 weeks of controlled refeeding after 2 weeks of semistarvation, is similar to that previously described in establishing a rat model for studying adjustments in energy expenditure specific for accelerating fat deposition during refeeding (Dulloo and Girardier, 1992; Crescenzo et al., 2003).

**Table 1:** **Composition of experimental diets rich in saturated-monounsaturated (SFA-MUFA) or polyunsaturated (PUFA) fatty acids**.

	**SFA-MUFA**	**PUFA**
Component, *g*		
Chow	57.0	57.0
Casein	13.1	13.1
Methionine	0.1	0.1
Choline	0.1	0.1
Vitamin mix	0.5	0.5
Mineral mix	1.8	1.8
Sunflower oil	1.4	1.4
Lard	26.0	−−−−−
Safflower oil	−−−−−	17.3
Linseed oil	−−−−−	8.7
Metabolisable energy, *kJ/100 g*	1885	1885
Gross energy, *kJ/100 g*	2150	2150
Macronutrients, *% ME*		
Protein	21.1	21.1
Lipid	58.2	58.2
Carbohydrate	20.7	20.7
Fatty acid composition, *g/100 g fatty acid*		
4:0-10:0	0.21	−−−−−
12:0	0.21	−−−−−
14:0	1.23	−−−−−
16:0	23.71	7.22
18:0	15.97	2.35
20:0	0.03	0.18
14:1n5	0.47	−−−−−
16:1n7	2.44	0.06
18:1n9	39.54	16.39
20:1n9	1.05	0.12
22:1n9	0.09	0.08
18:2n6	13.93	59.13
18:3n3	1.12	14.47
SFA%	41.4	9.8
MUFA%	43.6	16.6
PUFA%	15.0	73.6

At the end of refeeding period, all the rats were euthanized and blood was immediately collected for plasma preparation. Mesenteric and epididymal WAT (e-WAT) and interscapular BAT (i-BAT) were weighed and small aliquots of e-WAT and i-BAT were frozen for further measurements. Small aliquots of liver and hindleg skeletal muscle were also collected and used for measurements. The carcasses were finally used for body composition determination.

### Fuel oxidation and activity

Twenty-four hours VO2 and VCO2 of the rats were recorded with a four-chamber indirect open-circuit calorimeter (Panlab s.r.l., Spain). Measurements were performed every 15 min for 3 min in each cage. During the above measurements, continuous recording of spontaneous activity was also carried out through the extensiometric weight transducers located beneath each cage. Urine was collected for the whole (24-h) period and urinary nitrogen levels were measured by an enzymatic colorimetric method (FAR S.r.l., Italy). Substrate oxidation rates were calculated from VO2, VCO2 and urinary nitrogen according to Even et al. (1994).

### Plasma parameters

Rats were fasted for 6 h (8.00 a.m.–14.00 p.m.) and small blood samples were taken from the tail vein, placed in EDTA-coated tubes and transferred on ice. All the samples were then centrifuged at 1400 g_*av*_ for 15 min at 4°C, and then frozen. Plasma glucose concentration was measured by colorimetric enzymatic method (Pokler Italia, Italy), while plasma insulin concentration was measured using an ELISA kit (Mercodia AB, Sweden) in a single assay to remove inter-assay variations. Basal postabsorptive values of plasma glucose and insulin were used to calculate Homeostatic Model Assessment (HOMA) index as [Glucose (mg/dL) × Insulin (mU/L)]/405. HOMA index has been validated as an easy but accurate measure of insulin sensitivity in Wistar and Sprague-Dawley rats (Cacho et al., 2008). Plasma concentrations of triglycerides, cholesterol, non esterified fatty acids (NEFA), and alanine aminotransferase (ALT) were measured by colorimetric enzymatic method using commercial kits (SGM Italia, Italy and Randox Laboratories ltd., United Kingdom). Lipid peroxidation was determined according to Fernandes et al. (2006), by measuring thiobarbituric acid reactive substances (TBARS). Aliquots of plasma were added to 0.5 ml of ice-cold 40% trichloroacetic acid. Then, 1 ml of 0.67% of aqueous thiobarbituric acid containing 0.01% of 2,6-di-tert-butyl-p-cresol was added. The mixtures were heated at 90°C for 15 min, then cooled in ice for 10 min, and centrifuged at 850 g_*av*_ for 10 min. The supernatant fractions were collected and lipid peroxidation was estimated spectrophotometrically at 530 nm. The amount of TBARS formed was calculated using a molar extinction coefficient of 1.56 × 10^5^/M/cm and expressed as nmol TBARS/ml.

### Body composition and energy balance

The carcasses were incised and the gut cleaned of undigested food, then the whole carcasses were homogenized in distilled water, aliquoted, and frozen. Samples of the homogenized carcass were dried and analyzed for energy content by bomb calorimetry (Parr Instruments, IL, USA) and for fat content by the Folch extraction method (Folch et al., 1957). Body protein was determined from a general formula relating energy derived from fat, total energy value of the carcass, and energy derived from protein (Dulloo and Girardier, 1992); the caloric values for body fat and protein were taken as 38.6 and 22.7 kJ/g, respectively. Water, fat, and fat free dry mass were then calculated.

Energy balance measurements were conducted during refeeding by the comparative carcass technique over 2 weeks, during which metabolisable energy (ME) intake was monitored, as detailed previously (Iossa et al., 1997). Energy expenditure was determined as the difference between energy gain and ME intake, and the energetic efficiency was calculated as the percentage of total energy gain per ME intake.

### *De novo* lipogenesis in liver, e-WAT and i-BAT

Fatty acid synthase (FAS) activity was measured according to the protocol described by Penicaud et al. (1991) on protein extracts from liver, e-WAT and i-BAT. FAS activity was assessed in e-WAT since previous results have shown a downregulation of its activity in this specific fat pad in rats refed with SFA-MUFA diet (Marcelino et al., 2013).

### Triglyceride and glycogen content and lipid peroxidation in liver and skeletal muscle and hepatic myeloperoxidase (MPO) activity and TNF-α content

Tissue triglycerides were measured by colorimetric enzymatic method using commercial kits (SGM Italia, Italy). Tissue glycogen content was assessed by direct enzymatic procedure (Roehrig and Allred, 1974).

Lipid peroxidation was determined in liver and skeletal muscle by using the same procedure used for plasma samples. The amount of TBARS formed was expressed as nmol TBARS/g tissue.

The determination of MPO activity can be used as a surrogate marker of inflammation, since it has been shown that the activity of MPO solubilised from the inflamed tissue is directly proportional to the number of neutrophils seen in histologic sections (Krawisz et al., 1984). MPO activity was therefore assessed in liver samples as reported by Kim et al. (2012). Briefly, tissue samples (100 mg) were homogenized in 1 ml of hexadecyltrimethylammonium bromide (HTAB) buffer (0.5% HTAB in 50 mM phosphate buffer, pH 6.0) and centrifuged at 13,400 × g_av_ for 6 min at 4°C. MPO activity was measured spectrophotometrically: 10 μl of supernatant were combined with 200 μl of 50 mM phosphate buffer, pH 6.0, containing 0.167 mg/ml 0-dianisidine hydrochloride and 1.25% hydrogen peroxide. The change in absorbance at 450 nm was measured and one unit of MPO activity was defined as that degrading 1 μmol of peroxide per minute at 25°C.

TNF-α concentrations in protein extracts from liver were determined using a rat specific ELISA (R&D Systems, MN, USA) according to manufacturer's instruction. Briefly, the wells of a microtitre plate were coated with 100 μl of mouse anti-rat TNF-α (4 μg/ml) in PBS (137 mM NaCl, 2.7 mM KCl, 8.1 mM Na2HPO4, 1.5 mM KH2PO4, pH 7.4), and incubated overnight at room temperature. The antibody excess was then removed by washing with Wash Buffer (containing 0.05% (v/v) Tween 20 in PBS, pH 7.4), and the remaining sites on the plate were blocked with reagent diluent (PBS containing 1% BSA) (1 h, room temperature). After extensive washing, samples were added to the wells and incubated for 2 h at room temperature. After further washing, the wells were incubated with biotinylated goat anti-rat TNF-α (225 ng/ml in reagent diluent) followed by treatment with Streptavidin-HRP (1:200 dilution; 1 h, room temperature). Peroxidase-catalyzed color development from o-Phenylenediamine was measured at 492 nm.

### Oxidative capacities in mitochondria isolated from liver and skeletal muscle

Isolation of mitochondria from liver and skeletal muscle and measurements of respiration were carried out as previously reported (Iossa et al., 2003) and outlined below.

Tissue fragments from fresh liver were gently homogenized with a medium containing 220 mM mannitol, 70 mM sucrose, 20 mM HEPES, 1 mM EDTA, and 0.1% (w/v) fatty acid free bovine serum albumin (BSA), pH 7.4, in a Potter Elvehjem homogenizer set at 500 rpm (4 strokes/min). After withdrawn of aliquots for further assays, the homogenate was then centrifuged at 1,000 g_*av*_ for 10 min and the resulting supernatant was again centrifuged at 3,000 g_*av*_ for 10 min. The mitochondrial pellet was washed twice and finally resuspended in a medium containing 80 mM KCl, 50 mM HEPES, 5 mM Tris-PO_4_, 1 mM EGTA, 0.1% (w/v) fatty acid-free BSA, pH 7.0.

Freshly isolated hind leg muscles were freed of excess connective tissue, finely minced, washed in a medium containing 100 mM KCl, 50 mM Tris, pH 7.5, 5 mM MgCl_2_, 1 mM EDTA, 5 mM EGTA, and 0.1% (w/v) fatty acid free BSA, and treated with protease nagarse (1 mg/g) for 4 min. Tissue fragments were then homogenized with the above medium (1:8, w/v) at 500 rpm (4 strokes/min). The homogenate was centrifuged at 3,000 g for 10 min, the resulting supernatant was rapidly discarded and the pellet was resuspended and centrifuged at 500 g for 10 min. The supernatant was then centrifuged at 3,000 g for 10 min, the pellet was washed once and resuspended in a suspension medium (250 mM sucrose, 50 mM Tris, pH 7.5 and 0.1% fatty acid free BSA). Control experiments of enzymatic and electron microscopy characterization have shown that our isolation procedure (centrifugation at 3,000 g_*av*_ for 10 min) results in a cellular fraction, which is essentially constituted by mitochondria.

Oxygen consumption rate of isolated mitochondria was measured polarographically with a Clark-type electrode (Yellow Springs Instruments, OH, USA) in a 3 mL-glass cell, at a temperature of 30°C in a medium containing 80 mM KCl, 50 mM HEPES, 5 mM K_2_HPO_4_, 1 mM EGTA, 0.1% (w/v) fatty acid free BSA, pH 7.0, for liver mitochondria or 30 mM KCl, 6 mM MgCl_2_, 75 mM sucrose, 1 mM EDTA, 20 mM KH_2_PO_4_, pH 7.0, and 0.1% (w/v) fatty acid-free BSA, pH 7.0 for skeletal muscle mitochondria. The substrates used were 10 mM succinate + 3.75 μM rotenone, 10 mM glutamate + 2.5 mM malate, 40 μM palmitoyl-carnitine + 2.5 mM malate. All samples were allowed to oxidize their endogenous substrates for 3 min and then 10 mM succinate + 3.75 μM rotenone, 40 μM palmitoyl-carnitine + 2.5 mM malate, or 10 mM glutamate + 2.5 mM malate were added as substrate. State 3 oxygen consumption was measured in the presence of 0.3 mM ADP.

### Mitochondrial aconitase, lipid peroxidation and superoxide dismutase (SOD) specific activity

Active aconitase specific activity was measured spectrophotometrically by following the formation of NADPH (340 nm) at 25°C in a mixture containing 0.2 mM NADP^+^, 5 mM sodium citrate, 0.6 mM MnCl_2_, 1 U/mL concentration of isocitric dehydrogenase, 50 mM Tris-HCl, pH 7.4 (Gardner, 2002). Aconitase inhibited by ROS *in vivo* was reactivated so that total activity could be measured by incubating mitochondrial extracts in a medium containing 50 mM dithiothreitol, 0.2 mM Na_2_S, and 0.2 mM ferrous ammonium sulfate.

Lipid peroxidation was determined in isolated mitochondria by using the same procedure described before and was expressed as nmol TBARS/mg protein.

SOD specific activity was measured in a medium containing 0.1 mM EDTA, 2 mM KCN, 50 mM KH_2_PO_4_ pH 7.8, 20 mM cythocrome c, 0.1 mM xanthyne, and 0.01 units of xanthyne oxidase. Determinations were carried out spectrophotometrically (550 nm) at 25°C, by monitoring the decrease in the reduction rate of cythocrome c by superoxide radicals, generated by the xanthine-xanthine oxidase system (Flohè and Otting, 1974). One unit of SOD activity is defined as the concentration of enzyme that inhibits cythocrome c reduction by 50% in the presence of xanthine + xanthine oxidase.

### Western blot quantification of uncoupling protein 1 (UCP1) content in i-BAT

Tissue samples were homogenized in lysis buffer containing 20 mM Tris-HCl (pH 7.5), 150 mM NaCl, 2.7 mM KCl, 5% (v/v) glycerol, 1% (v/v) Triton X-100, and 50 μL/g tissue of protease inhibitor cocktail (all from Sigma-Aldrich, MO, USA) using a Potter homogeniser, shaken for 2 h at 4°C, and centrifuged at 14,000 g_*av*_ for 20 min at 4°C. The supernatants were collected, aliquots were denatured in a buffer (60.0 mM Tris pH 6.8, 10% sucrose, 2% SDS, 4% β-mercaptoethanol) and loaded onto a 12% SDS-Polyacrylamide gel. After the run in electrode buffer (50 mM Tris, pH 8.3, 384 mM glycine, 0.1% SDS), the gels were transferred onto polyvinylidene difluoride membranes (Immobilon-P, Merck Millipore, Germany) at 0.8 mA/cm^2^ for 90 min. The membranes were preblocked in blocking buffer (PBS; 5% milk powder; 0.5% Tween 20) for 1 h and then incubated overnight at 4°C with a rabbit antibody for UCP1 (Alpha Diagnostic International, TX, USA, at 1 μg/mL dilution in blocking buffer). The membranes were washed and then incubated for 1 h at room temperature with an anti-rabbit alkaline phosphatase-conjugated secondary antibody (Promega, WI, USA). The membranes were finally washed, rinsed in distilled water and incubated at room temperature with a chemiluminescent substrate, CDP-Star (Sigma-Aldrich, MO, USA). Data detection was carried out by exposing autoradiography films (Eastman Kodak Company, NY, USA) to the membranes. Quantification of signals was carried out by Un-Scan-It gel software (Silk Scientific, UT, USA). Actin was detected with polyclonal antibody (Sigma-Aldrich, MO, USA diluted 1:250 in blocking buffer) and used to normalize the UCP1 signal.

### Statistical analysis

Data are given as means with their standard errors. Statistical analyses were performed by two-tailed, unpaired, Student's *t*-test. Probability values less than 0.05 were considered to indicate a significant difference. All analyses were performed using GraphPad Prism 6 (GraphPad Software, CA, USA).

### Materials

All chemicals used were of analytical grade and were purchased from Sigma-Aldrich (MO, USA).

## Results

Body composition analysis revealed that rats refed the PUFA-enriched diet had lower body lipids and higher body proteins compared to rats refed the SFA-MUFA-enriched diet (Table 2). In addition, rats refed PUFA diet had lower amount of visceral (mesenteric and epididymal) WAT, but higher amount of i-BAT compared to rats refed SFA-MUFA diet (Table 2). The different final body composition was the result of a different lipid and protein gain during the 2-weeks refeeding period. In fact, rats refed PUFA diet gained significantly less lipids and more proteins compared to those refed SFA-MUFA diet (Table 2).

**Table 2:** **Body composition and energy balance in rats refed diet rich in saturated-monounsaturated (SFA-MUFA) or polyunsaturated (PUFA) fatty acids**.

	**SFA-MUFA**	**PUFA**
Final body weight, g	370 ± 6	368 ± 7
Body weight gain, g	127 ± 9	128 ± 8
Body lipids, %	15.4 ± 0.5	13.8 ± 0.3^*^
Body proteins, %	14.0 ± 0.3	15.4 ± 0.3^**^
Epididymal white adipose tissue weight, g/100 g b.w.	1.47 ± 0.06	1.20 ± 0.06^**^
Mesenteric white adipose tissue weight, g/100 g b.w.	0.12 ± 0.01	0.08 ± 0.01^*^
Interscapular brown adipose tissue weight, g/100 g b.w.	0.099 ± 0.01	0.135 ± 0.01^*^
Body energy, kJ/g	9.3 ± 0.2	9.0 ± 0.2
ME intake, kJ	5354 ± 76	5325 ± 119
Energy gain, kJ	1888 ± 127	1715 ± 98
Lipid gain, kJ	1633 ± 98	1362 ± 53^*^
Protein gain, kJ	255 ± 12	353 ± 13^**^
Energy expenditure, kJ	3466 ± 102	3610 ± 80
Metabolic efficiency, %	35.3 ± 2.6	32.2 ± 2.5

*Values are reported as means with their standard errors. N = 8 different rats*.

* *P < 0.05 compared to SFA-MUFA*.

** *P < 0.01 compared to SFA-MUFA*.

*ME = metabolisable energy; b.w. = body weight*.

At the end of the refeeding period, indirect calorimetry measurements were performed to evaluate the differences in the oxidation of macronutrients. Rats refed PUFA diet exhibited higher NPRQ values and non-protein energy utilization was fulfilled by using proportionally more carbohydrates and less fat compared to rats refed SFA-MUFA diet (Table 3). In addition, evaluation of the mixture of oxidized fuels as a proportion of energy expenditure showed that in rats refed PUFA diet, the utilization of proteins as energy fuels was lower, while the utilization of carbohydrates and lipids was higher compared to rats refed SFA-MUFA diet (Table 3). No significant variation was found in the activity levels of the two groups of rats (Table 3).

**Table 3:** **Substrate balance and daily spontaneous activity in rats refed diet rich in saturated-monounsaturated (SFA-MUFA) or polyunsaturated (PUFA) fatty acids**.

	**SFA-MUFA**	**PUFA**
NPRQ	0.79 ± 0.01	0.83 ± 0.01^*^
%Carbohydrates	29 ± 2	42 ± 4^*^
%Lipids	71 ± 5	58 ± 3^*^
%energy expenditure coming from:		
Lipids	38.6 ± 2.0	49.8 ± 2.0^**^
Proteins	38.2 ± 2.0	17.1 ± 1.0^**^
Carbohydrates	23.2 ± 2.0	33.1 ± 3.0^*^
RQ	0.79 ± 0.01	0.78 ± 0.01
Daily spontaneous activity, arbitrary units	728 ± 23	713 ± 25

*Values are reported as means with their standard errors. N = 8 different rats*.

* *P < 0.05 compared to SFA-MUFA*.

** *P < 0.01 compared to SFA-MUFA*.

*RQ = respiratory quotient, NPRQ = non protein respiratory quotient*.

Rats refed PUFA diet had lower HOMA index and plasma insulin levels in the fasting and the fed state, together with lower plasma triglycerides and cholesterol levels than rats refed SFA-MUFA diet (Table 4). Plasma lipid peroxidation was not significantly different between the two groups of rats, while a significant decrease in ALT was found in rats refed PUFA diet compared to those refed SFA-MUFA diet (Table 4).

**Table 4:** **Plasma parameters in rats refed diet rich in saturated-monounsaturated (SFA-MUFA) or polyunsaturated (PUFA) fatty acids**.

	**SFA-MUFA**	**PUFA**
Fasting glucose, mg/dl	115.6 ± 3.0	124.1 ± 2.8
Fasting insulin, mU/l	14.2 ± 0.5	9.8 ± 1.1^**^
HOMA index	4.0 ± 0.1	3.0 ± 0.1^**^
Postprandial glucose, mg/dl	343 ± 20	358 ± 22
Postprandial insulin, mU/l	89 ± 4	56 ± 2^**^
Postprandial insulin/fasting insulin	6.3 ± 0.2	5.6 ± 0.2^*^
Lipid peroxidation, nmol TBARS/ml	14.3 ± 15.8	15.8 ± 1.4
NEFA, mmol/l	1.9 ± 0.2	1.8 ± 0.2
Triglycerides, mg/dl	275 ± 21	185 ± 13^**^
Cholesterol, mg/dl	142 ± 6	122 ± 5^*^
ALT, U/l	13.9 ± 1.0	9.4 ± 1.0^**^

*Values are reported as means with their standard errors. N = 8 different rats*.

* *P < 0.05 compared to SFA-MUFA*.

** *P < 0.01 compared to SFA-MUFA*.

*HOMA = Homeostatic Model Assessment; HOMA index = Glucose (mg/dl) × Insulin(mU/l)/405; NEFA = non-esterified fatty acids; TBARS = thiobarbituric acid reactive substances; ALT = alanine aminotransferase*.

**Figure 1:**
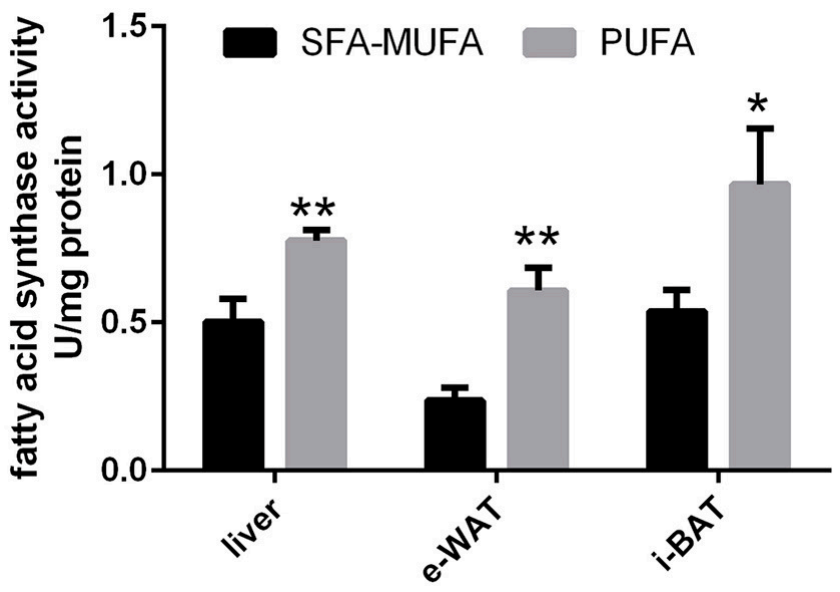
**Fatty acid synthase activity in liver, epididymal-WAT (e-WAT) and interscapular-BAT (i-BAT) in rats refed diet rich in saturated-monounsaturated (SFA-MUFA) or polyunsaturated (PUFA) fatty acids**. Values are reported as means with their standard errors. *N* = 8 different rats. ^*^P < 0.05, ^**^P < 0.01 compared to SFA-MUFA.

FAS activity, the rate-limiting enzyme in the pathway of *de novo* lipogenesis, was found to be significantly higher in liver (+50%, *p* < 0.01), e-WAT (>2-folds, *p* < 0.01) and i-BAT (nearly 2-folds, *p* < 0.05) from rats refed PUFA diet compared to those refed SFA-MUFA diet (Figure 1).

**Figure 2:**
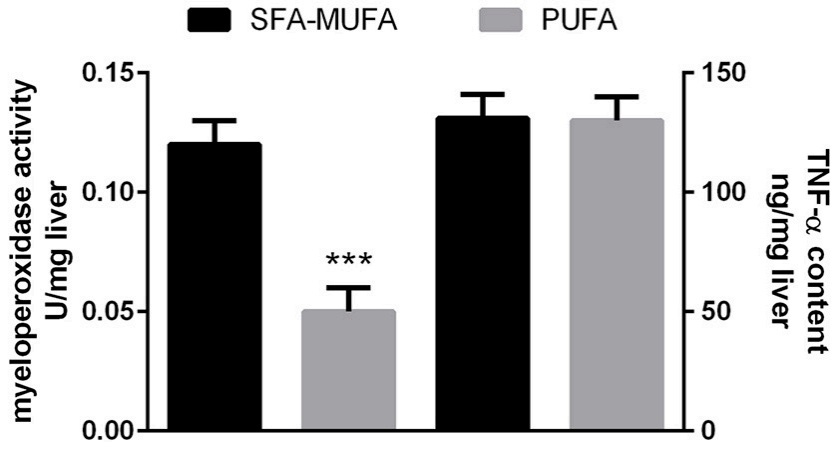
**Myeloperoxidase activity and TNF-α content in liver from rats refed diet rich in saturated-monounsaturated (SFA-MUFA) or polyunsaturated (PUFA) fatty acids**. Values are reported as means with their standard errors. *N* = 8 different rats. ^***^P < 0.001 compared to SFA-MUFA.

In the liver, higher triglyceride content was found in rats refed PUFA diet, while no such difference was found in skeletal muscle (Table 5). Hepatic lipid peroxidation was significantly lower in rats refed PUFA diet, while no difference was found in skeletal muscle (Table 5). No significant variation was found in glycogen content in liver and skeletal muscle (Table 5). Degree of hepatic inflammation was evaluated by measuring (i) the activity of MPO (an enzyme produced in leukocytes and whose activity is linearly related to neutrophil infiltration of the tissues) as an index of inflammatory response under pathological conditions (Villegas et al., 2003), together with (ii) hepatic content of the proinflammatory mediator TNF-α. The results indicate that MPO activity was significantly lower (by ~60%, p < 0.001) in rats refed PUFA diet compared to those SFA-MUFA diet, while the two groups of rats exhibited no significant difference in hepatic content of TNF-α (Figure 2).

**Table 5:** **Liver and skeletal muscle composition in rats refed diet rich in saturated-monounsaturated (SFA-MUFA) or polyunsaturated (PUFA) fatty acids**.

	**SFA-MUFA**	**PUFA**
**LIVER**		
Triglycerides, mg/g	24.2 ± 1.8	40.5 ± 2.8^**^
Lipid peroxidation, nmol TBARS/g	92 ± 5	64 ± 3^**^
Glycogen, mg/g	40 ± 2	38 ± 2
**SKELETAL MUSCLE**		
Triglycerides, mg/g	3.0 ± 0.1	2.7 ± 0.1
Lipid peroxidation, nmol TBARS/g	42 ± 2	45 ± 3
Glycogen, mg/g	58 ± 3	56 ± 4

Values are reported as means with their standard errors. N = 8 different rats. ^*^P < 0.05 compared to SFA-MUFA.

** *P < 0.01 compared to SFA-MUFA*.

*TBARS = thiobarbituric acid reactive substances*.

**Figure 3:**
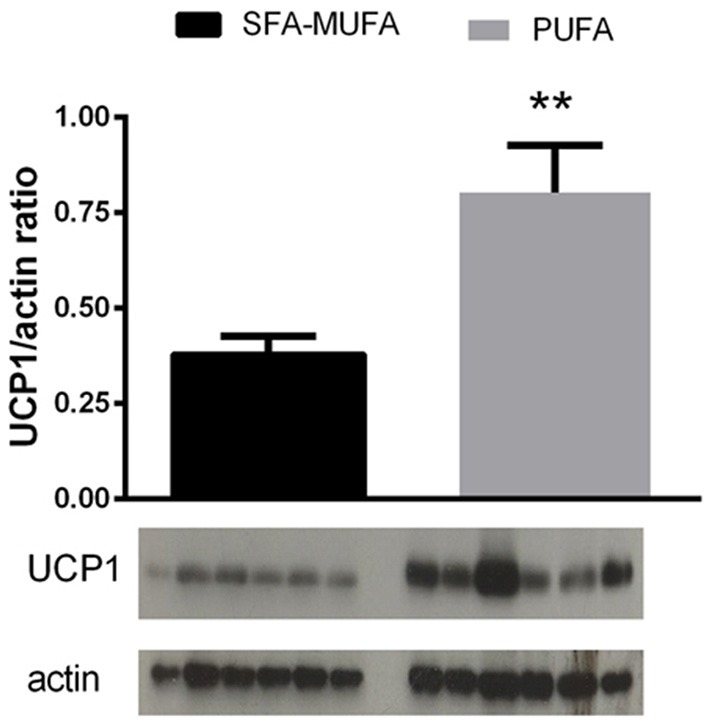
**Uncoupling protein (UCP) 1 content in interscapular BAT from rats refed diet rich in saturated-monounsaturated (SFA-MUFA) or polyunsaturated (PUFA) fatty acids**. Values are reported as means with their standard errors. *N* = 6 different rats. ^**^P < 0.01 compared to SFA-MUFA.

Expression of the UCP-1 protein in i-BAT was found significantly increased (by 2-folds, *p* < 0.01) in rats refed PUFA diet (Figure 3).

No significant differences in mitochondrial respiratory capacities and oxidative damage to lipids and proteins in liver and skeletal muscle were found between rats refed SFA-MUFA or PUFA diet (Tables 6, 7). In addition, SOD activity was found to be significantly higher in liver mitochondria from rats refed PUFA diet compared to those refed SFA-MUFA diet, while no difference was found in skeletal muscle mitochondria (Table 7).

**Table 6:** **Mitochondrial respiratory capacities in liver and skeletal muscle from rats refed diet rich in saturated-monounsaturated (SFA-MUFA) or polyunsaturated (PUFA) fatty acids**.

	**SFA-MUFA**	**PUFA**
**LIVER**		
Glutamate+malate	100 ± 7	126 ± 6
Palmitoyl-carnitine+malate	97 ± 7	112 ± 7
Succinate+rotenone	171 ± 11	194 ± 10
**SKELETAL MUSCLE**		
Glutamate+malate	742 ± 43	729 ± 33
Palmitoyl-carnitine+malate	337 ± 43	374 ± 45
Succinate+rotenone	723 ± 23	657 ± 20

*Values are expressed as ngatoms of oxygen/(min × mg protein) and reported as means with their standard errors. N = 8 different rats*.

**Table 7:** **Mitochondrial oxidative status in liver and skeletal muscle from rats refed diet rich in saturated-monounsaturated (SFA-MUFA) or polyunsaturated (PUFA) fatty acids**.

	**SFA-MUFA**	**PUFA**
**HEPATIC MITOCHONDRIA**		
Active aconitase, mU/mg protein	4.4 ± 0.4	5.3 ± 0.5
Total aconitase, mU/mg protein	11.2 ± 0.3	11.0 ± 0.6
Active/total aconitase ratio	0.40 ± 0.04	0.48 ± 0.03
Lipid peroxidation, nmol TBARS/mg protein	0.32 ± 0.01	0.31 ± 0.01
SOD, U/mg protein	78 ± 7	100 ± 7^*^
**SKELETAL MUSCLE MITOCHONDRIA**		
Active aconitase, mU/mg protein	15.3 ± 1.1	18.9 ± 1.1^*^
Total aconitase, mU/mg protein	32.8 ± 1.8	40.6 ± 1.5^**^
Active/total aconitase ratio	0.47 ± 0.029	0.47 ± 0.018
Lipid peroxidation, nmol TBARS/mg protein	3.4 ± 0.1	3.6 ± 0.2
SOD, U/mg protein	137 ± 8	135 ± 6

*Values are reported as means with their standard errors. N = 8 different rats*.

* *P < 0.05*,

** *P < 0.01 compared to SFA-MUFA*.

*SOD = superoxide dismutase, TBARS = thiobarbituric acid reactive substances*.

## Discussion

In the present study, we provide evidence that during catch-up fat upon refeeding with high fat diets, the presence of dietary PUFA not only prevents excessive fat deposition and stimulates protein deposition but also enhances *de novo* lipogenesis in liver, e-WAT, and i-BAT, thus increasing glucose utilization in these tissues and improving glucose homeostasis. Despite similar total body energy gain, rats refed with PUFA-enriched diet stored less energy as body lipid in favor of protein deposition. The differences found in energy balance arise from divergent substrate balance between the two groups of rats. In fact, despite the same RQ value, rats fed PUFA diet rely less on protein oxidation and more on carbohydrate and lipid oxidation to support daily energy expenditure, compared to rats refed SFA-MUFA diet. Furthermore, non-protein substrate balance indicated that in rats refed PUFA diet, proportionally more carbohydrates and less fat is oxidized to produce energy. The higher carbohydrate oxidation found in rats refed PUFA diet confers a greater capacity to maintain glucose homeostasis, as also indicated by a lower HOMA index and plasma insulin levels both in the fasted and in the fed state.

The higher oxidation of glucose elicited by refeeding with PUFA diet reflects changes in metabolic fluxes in organs and tissues that greatly influence carbohydrate balance. For this reason, we looked at liver, skeletal muscle, WAT and BAT which, via different mechanisms, play a role in glucose homeostasis (Rosen and Spiegelman, 2006; Stanford et al., 2013). In liver and skeletal muscle, mitochondrial oxidative capacity was found unchanged with the high PUFA diet, thus ruling out the possibility of an increased use of glucose as fuel by these organelles. Similarly, the unchanged glycogen content of these two tissues also excludes an increase in glucose usage for storage. In liver, as well as in WAT and BAT, another primary route of glucose utilization is the pathway of *de novo* lipogenesis. Indeed, when rats are refed a low fat diet, *de novo* lipogenesis has been shown to be increased in liver and e-WAT (Crescenzo et al., 2010; Marcelino et al., 2013), and it has been proposed that utilization of glucose in this metabolic pathway helps maintenance of glucose homeostasis while ensuring a rapid replenishment of body fat reserves (Marcelino et al., 2013). Conversely, in rats refed with a SFA-MUFA diet, *de novo* lipogenesis is markedly blunted in liver and e-WAT (Crescenzo et al., 2012; Marcelino et al., 2013), thereby contributing to the derangement of glucose homeostasis. Consistent with this contention are our findings here that the improvement in glucose homeostasis during refeeding on the high PUFA diet is associated with an enhanced activity, in e-WAT, i-BAT, and liver, of FAS -the rate limiting enzyme of the biosynthetic pathway of *de novo* lipogenesis (including neogenesis of fatty acids from glucose). Therefore, the inhibition of *de novo* lipogenesis previously found using a SFA-MUFA high fat diet (Crescenzo et al., 2012; Marcelino et al., 2013) may at least partially be prevented by PUFA rich diet. The high rates of *de novo* lipogenesis in all three above tissues could represent a relevant buffering system that would contribute to the glucose homeostasis. In addition, since *de novo*-derived lipids imply a higher energetic cost of deposition compared to diet-derived lipids, the upregulation of *de novo* lipogenesis contributes to burn more calories and limit the deposition of body fat. An increase in UCP1 expression in i-BAT was also shown in the group of rats refed with PUFA-enriched diet. It is known that the brown adipocyte, whose activation is sympathetically controlled, owes its thermogenic capacity to the presence of UCP1. This inner mitochondrial protein is able to dissociate oxidative phosphorylation from ATP synthesis (Chechi et al., 2013) and its expression is physiologically enhanced by adrenergic stimulation (Chechi et al., 2013). This result strongly supports the contention that the lower body fat accretion, occurring with PUFA-enriched diet, is not only the result of increased partitioning to lean mass, but could be also ascribed to an increased thermogenic stimulation of BAT. Based upon the high energy cost of protein deposition and its maintenance compared to the low energy cost of fat deposition and maintenance on high fat diets, it can be calculated that of the lower fat accretion observed in rats fed a PUFA-enriched diet, about 80% can be attributed to an increased partitioning to fat-free mass and the associated cost of storage, and about 20% to increased thermogenesis.

Interestingly, also from the analysis of plasma parameters, we found that the levels of triglycerides and cholesterol decrease significantly in rats fed a PUFA-enriched diet, thus indicating that also circulating lipids are reduced after refeeding with this diet.

The increased hepatic *de novo* lipogenesis in rats refed PUFA diet could lead to increased triglyceride deposition in this tissue. In fact, livers from rats fed PUFA diet display increased triglyceride content, similarly to what we previously reported after refeeding with a safflower oil-rich high fat diet (Crescenzo et al., 2012). Accumulation of triglycerides within the liver was thought to represent the “first-hit” in the progression of nonalcoholic fatty liver disease. However, it has been suggested that the ability to synthesize triglycerides constitutes a protective mechanism, limiting the evolution of liver injury and fibrosis (Choi and Diehl, 2008; Solinas et al., 2015). In fact, if hepatocyte triglyceride synthesis is inhibited, free fatty acids accumulate in the liver, leading to induction of fatty acid oxidizing systems that increased hepatic oxidative stress and liver damage (Choi and Diehl, 2008; Solinas et al., 2015). Consequently, the ability of the liver to synthesize triglycerides may be protective in conditions of excess availability of fatty acids. In agreement with this view, we have found here a lower oxidative stress, degree of inflammation and necrosis in liver from rats refed PUFA diet. These results thus suggest the lack of the “second-hit” that is needed for the progression of simple steatosis versus nonalcoholic fatty liver disease.

In conclusion, when considering the composition of high fat diets for nutritional rehabilitation, the inclusion of PUFA could be useful for improving protein deposition and maintaining glucose homeostasis, while limiting lipid storage in adipose tissue and oxidative stress and inflammation in the liver.

## Author contributions

RC, GL, AD, SI conceived the study; all the authors designed the experiments; RC, AM, RCa, FB, and AG performed the experiments; RC, AM, RCa, FB, AG, GL, SI analyzed the data and performed the statistical analyses; RC, GL, SI drafted the manuscript and all authors contributed in the revision, gave final approval for publication and agreed to be accountable for all aspects of the work in ensuring that questions related to the accuracy or integrity of any part of the work are appropriately investigated and resolved.

## Funding

This work was supported by grant from University “Federico II” of Naples, and by the Swiss National Science Foundation (grant no. 310030-152870).

### Conflict of interest statement

The authors declare that the research was conducted in the absence of any commercial or financial relationships that could be construed as a potential conflict of interest.
